# Hooked on virtual social life. Problematic social media use and associations with mental distress and addictive disorders

**DOI:** 10.1371/journal.pone.0248406

**Published:** 2021-04-08

**Authors:** Vincent Henzel, Anders Håkansson

**Affiliations:** 1 Faculty of Medicine, Dept of Clinical Sciences Lund, Psychiatry, Lund University, Lund, Sweden; 2 Malmö Addiction Center, Region Skåne, Malmö, Sweden; University of Auckland, NEW ZEALAND

## Abstract

**Background:**

Social media is an important and growing part of the lives of the vast majority of the global population, especially in the young. Although still a young and scarce subject, research has revealed that social media has addictive potential. The aim of this cross-sectional study was to explore the associations between problematic use of social media and mental distress, problematic gaming and gambling, within the Swedish general population.

**Methods:**

Data from 2,118 respondents was collected through self-report questions on demographics and validated scales measuring addiction-like experiences of social media, problem gaming, problem gambling, and mental distress. Associations were analyzed in unadjusted analyses and–for variables not exceedingly inter-correlated–in adjusted logistic regression analyses.

**Results:**

In adjusted analyses, problematic use of social media demonstrated a relationship with younger age, time using instant messaging services, and mental distress, but not with education level, occupational status, or with treatment needs for alcohol or drug problems. Behavioral addictions (internet, gaming and gambling) were substantially inter-correlated, and all were associated with problematic use of social media in unadjusted analyses.

**Conclusions:**

Social media use is associated with other addictive behaviors and mental distress. While factors of causality remain to be studied, these insights can motivate healthcare professionals to assess social media habits, for example in individuals suffering from issues concerning gambling, gaming or mental health.

## Introduction

There is a growing body of research indicating that using modern technologies, such as the Internet, video games, smart phones and social media platforms, has the potential of being addictive [1–3]. Social media addiction is not yet an established diagnosis, although it is one of many suggested behavioral addictions [4]. Among these, the gambling disorder is the only one recognized in the Diagnostic and Statistical Manual for Mental Disorders, while Internet gaming disorder is listed in its appendix as a condition which requires further research [5].

Worldwide, the number of people with an active Facebook account is predicted to surpass three billion [6] in the year 2021. Other popular platforms such as YouTube, Snapchat, and Instagram are also attracting an increasing number of users, especially from younger populations [7]. Constant online presence and availability has become something of a status quo in the lives of the vast majority, made possible with the rise of highly accessible and user-friendly laptops and smartphones. Using social media is one of the most common activities on the Internet [8], with daily use reported by nine out of ten youths worldwide [9]. Social media presents users with a broad spectrum of activities, ranging from maintenance of real life relationships through chats and calls, sharing one’s own or others’ creative content and opinions, partaking in communities, playing games, gambling, and passing time looking through the activities of other users [3]. This diversity of activities is a scientific challenge concerning whether social media can be considered addictive as a whole, or if it is rather a question of which parts of it that are potentially negative, as well as beneficial for the individual.

Academic work in this field has been criticized for its inconsistencies of defining normal and problematic usage, as well as using non-standardized measurement tools, subsequently rendering comparison of results and prevalence rates difficult. Confusion also surrounds what social media actually entails, and whether it is synonymous to social networking or not, as it is sometimes referred as [4, 10]. In fact, the existent literature is inconsistent on labeling this issue; social media addiction, excessive, problematic, and at-risk use are applied both separately and homogeneously. Moreover, online activities such as sharing photos, engaging in communities, and communicating with real life friends can be associated with lower degree of loneliness and less psychological distress [11, 12]. These particular activities are also believed to be mediators of shaping and maintaining the user’s sense of identity and belonging, safety and competence, satisfying basic psychological needs [13], as well as bringing higher psychosocial wellbeing, which apparently is the case particularly for young users [14–16].

Studies have demonstrated that interrelationships between different behavioral addictions exist, suggesting common underlying risk factors, together with associations with addictive use of psychoactive substances [17]. This has bred a widely used matrix of characteristics, first proposed by Griffiths as a ‘components model of addiction’ [18], involving a number of key components; salience (typically defined as the preoccupation in thoughts, and the vast amount of time and energy spent on either thinking of or carrying out the behavior), mood modification, tolerance, withdrawal, loss of control, and conflicts [18, 19]. Some authors [20, 21] have proposed socio-cognitive mechanisms for how Internet-related addictions develop. These revolve around negative beliefs about the self and the world, with thoughts such as ‘I’m nobody offline, but online I am someone’ or ‘the online world is safer than the real one’. This could reasonably lead to a preference for online interaction, an overreliance to the services provided by the Internet and social media, thus giving rise to an addictive state for the individual. With the component model as background, problematic use of social media has been suggested to consist of an exaggerated concern about social media, a strong motivation to use it, and the devotion of time and effort to social media to the extent that it negatively impacts on other social activities, studies, work, interpersonal relationships, and/or mental health and well-being [22].

There is a lack of reviews on the subject, and agreed-upon definitions or gold standard measurements and cutoffs have not been established. One part of this research gap is that–apart from differing definitions–prevalence rates reported also derive from different populations and in very different settings; prevalence figures reported have differed widely from a low of 1.6% in a study carried out in Nigeria, and a high of 34% in a study from China. When including general Internet addiction as well as Facebook addiction specifically, rates of 2–12% have been reported from various literature reviews [23–25]. Even though anyone with access to social media can become addicted to it, this holds true especially for young people, a group that has been increasingly exposed to technology, and consistently reported to use and abuse social media to a larger degree than adults [26]. From Europe and the US [27, 28], recent studies and reports have found high rates of technology use among the young. A large proportion of children as young as 8 months use a screen daily, with the majority of 11-year-olds owning a smartphone, and 8-12-year-olds using screens for entertainment purposes almost five hours per day.

There are also associations to being single, as well as to low education level and lower monthly income, and although evidence is somewhat inconclusive for gender differences, several findings point to a slight overrepresentation among female users [29–31]. Suffering from mental disorders such as depression and anxiety has been long known to increase the risk for, and be exacerbated by, behavioral and substance addictions alike [32, 33]. Numerous studies, including comprehensive reviews, have shown that addictive use of social media is associated with depression, anxiety and mental distress [26, 34–37].

There have been discoveries regarding associations and interrelationships between addictive online behavior and use of psychoactive substances, in a number of European countries. Problematic use of video games and social media have displayed a small but significant relationship [23], while multidirectional associations between problematic gaming, gambling, and internet use have been observed as well [38]. Excessive use of the Internet has been shown to increase likelihood for use [39–42] and abuse [43, 44] of alcohol and other substances. A relationship between time spent on social media and episodic heavy drinking has also been found [45].

There is a scarcity of studies on prevention or treatment for technology-related addictions. Mindfulness techniques [46], and psychotherapeutic interventions, have shown improvement of symptoms in some patients, but more controlled trials are needed to develop any standardized treatment regimens [47–49]. Thus, altogether, the overall understanding of addictive social media behavior is hitherto limited, with respect to topics ranging from risk factors and prevalence to the clinical picture and treatment possibilities. Based on this research gap, more knowledge is needed in the basic understanding of the existence and correlates of this construct in the population.

Altogether, based on the relative novelty of research in the area of social media addiction, large research gaps remain in the area. For example, considerably more information is needed about the correlates of problematic social media use, both in the population as a whole and in younger individuals specifically. Also, given the uncertainty so far about the definition of social media, there is reason to study if and how the present construct is association with other behavioral addictions often related to online platforms, such as gaming, gambling and the overall use of the internet. In addition, it is of importance to study and to control for the time spent in typically occurring instant messaging services, when also considering other types of possible risk factors.

Thus, the present study aimed to assess problematic use of social media as the primary outcome of the study, and exemplifying it mentioning some brand names representing the most common social media reported (Facebook and Instagram) [50], as well as one of the social media somewhat less common but often referred to in the media (Twitter). Also, the study aimed to outline the possible correlations of problematic social media use with demographics, mental distress, and other behavioral addictions, in the general population as a whole, as well as in the sub-population of adolescents/younger adults. The present study hypothesized that addictive social media behavior, in the general population, may be related to symptoms of psychological distress, and to a history of treatment needs for problematic alcohol or drug use.

## Methods

### Study design

A cross-sectional self-reported online survey design was utilized, targeting the general Swedish population. The questionnaire assessed a spectrum of behavioral addictions, of which problematic use of social media was the main outcome variable. In the present analyses, problematic social media use was treated as the outcome variable, testing it against a number of factors suggested in the literature to be associated with this behavior.

### Participants and procedures

Data collection from a general Swedish population sample was conducted with the help of a marketing survey company (Userneeds AB), who administered a self-report online questionnaire via e-mail to their web panel members in six age groups ranging from 16 to 80 years, aiming for a nationally representative sample. Potential participants included members of the web panel of Userneeds, who typically receive offers to participate in market surveys for commercial products and similar. Each completed survey grants the respondent a reward of approximately 1€.

Electronic written consent was required in order for the questionnaire to open. To ensure anonymity and confidentiality, the IP-address of each respondent was hidden from the researchers. To complete and thereby send the filled questionnaire to the researchers, every single question had to be answered, and though they could not be skipped, some items had an optional answer reading “do not wish to answer”. The target was to reach a sample of 2000 individuals, equally divided regarding gender, and stratified by age. The full sample was reached in October, 2019, totaling 2118 partial and 2002 completed surveys, ending four weeks of data collection.

The National Ethical Review Board, Sweden, approved the study in August, 2019, and stated it was not subject to Swedish ethics legislation as it does not involved identified personal data. The present study is based on data from the same overall online survey data collection as a different scientific publication in a separate line of research, a paper assessing history of voluntary self-exclusion in gambling through a novel multi-operator self-exclusion service in the present setting [51].

### Instruments and measures

For the primary outcome measure of social media addiction, the study used the *Bergen Social Media Addiction Scale* (BSMAS) includes six Likert scale items, graded 1–5 (‘Never’–‘Very often’) about the following experiences, during the last 12 months: spending a lot of time thinking of social media or planning what to do there; desiring to use social media more and more; using social media to forget about personal issues; tried to cut down use without success; becomes restless or anxious when unable to access social media; used social media to such a degree that it has impacted your work or studies negatively. Scoring ranges from 6–30, with 19 or above indicating problematic use of social media, and the scale has been validated and shown to have a Cronbach alpha of 0.86 [52, 53]. As in recent years, the authors behind the present scale have recommended a score of 19 as the cut-off indicating problematic social media use [52–54], the present study used a dichotomous classification of respondents as either above or below cut-off, rather than using the instrument in a dimensional way.

The start of the social media item was that the questions were to deal with the respondent’s use of social media (Facebook, Twitter, Instagram and similar) during the past 12 months. The social media given as examples here, as well as the ‘and similar’ wording, were provided in order to mention the most common ones, i.e. Facebook and Instagram, as well as Twitter as an example of a service also being relatively common but which may also appear in somewhat different contexts, such as in reports in the professional life and in business and traditional media [50].

In order to study potential correlates of social media addiction, related to problematic internet use, problem gambling, problem gaming and psychological distress, the study used the following screening tools:

1. The *Problematic and Risky Internet Use Screening Scale-3* (PRIUSS), is made up of three items grading Internet behavior with a five-point Likert scale ranging from never to very often. This scale has no time frame, rather it assesses current symptoms. The questions evaluated to what degree the subject had experienced increased anxiety because of Internet use, felt anxious when not using the Internet, and how often they felt a loss of motivation for more important tasks. Score range is 0–12, with a cutoff at 9 points for risky Internet use [55]. Cronbach-alpha value has been reported to be 0.81.
2. The *Gaming Addiction Scale* (GAS), also a five-point Likert scale ranging from 1–5 (‘Never’ to ‘Very often’), was developed and validated by Lemmens et. al [56]. It begins with the phrase ‘How often during the last six months…’, followed by seven questions: did you think about playing a game all day long; did you spend an increasing amount of time on games; did you play games to forget about real life; have others unsuccessfully tried to reduce your game use; have you felt bad when you were unable to play; did you have fights with others (e.g., family, friends) over your time spent on games, and; have you neglected other important activities(work, school, exercise) to play games. The cutoff for at-risk gaming behavior is 21 out of a total of 35 points, and the internal validity of this tool has repeatedly demonstrated a Cronbach-alpha above 0.7 [57].
3. The *Problem Gambling Severity Index* (PGSI), developed in Canada [58], consists of nine items corresponding to the diagnostic criteria for Gambling Disorder in the DSM-5. Assessing gambling behavior during the recent twelve month period, the scale grades loss of control, increased urges, returning to reclaim losses, whether they had borrowed money or sold something to gamble, self-awareness of problematic gambling, receiving criticism from others, feelings of guilt, gambling had caused any health issues, and lastly any financial problems. In this research paper, which does not seek to screen for or subdivide gambling behavior, scoring 9 or higher out of 27 possible points is regarded problematic [59, 60]. The Cronbach-alpha has been shown to be 0.77 [60].
4. The *Kessler Psychological Distress Scale* (Kessler-6), a six-item questionnaire with a 5-point Likert scale on each one (‘Not at all’ to ‘Almost constantly’), assessing non-specific mental distress during the past six months. It is widely used both clinically and academically as a tool for severity or screening [61]. With questions inspired from the symptomatology of depression and anxiety, it asks how often the respondent had been nervous, restless or fidgety, so depressed that nothing could cheer him/her up, felt that everything was an effort, and had felt worthlessness. A cut-off at 13 out of 24 possible points indicates severe mental distress. It includes an option to answer ‘Do not wish to answer’ on each item, although these were excluded from statistical analyses. The Cronbach-alpha of the Kessler-6 scale has been reported to be 0.89 [61]

In addition, study variables included sociodemographic data; age (in age groups), gender (male, female, or do not wish to answer), marital status, occupational status, level of education, and monthly income in discrete intervals. The five categories of occupational status were merged into two categories; working (59.8%), or not working (40.2%) which covered studying, seeking employment, being retired, or being on sick leave (Table 1).

**Table 1:** Description of the study sample. All individuals included (N = 2,002).

*Age(years)*	%(n)
16–19	3.2(64)
20–24	5.9(118)
25–29	9.6(192)
30–39	18.1(362)
40–49	24.5(490)
50 and above	38.8(776)
*Gender*	
Female	50.4(1010)
Male	49.5(990)
Does not identify	0.1(2)
*Occupational status*	
Working	59.8(1197)
Not working	40.2 (805)
*Education level*	
Primary School	5.5(111)
High School	34.7(695)
Incomplete university degree	16.6(333)
University Degree	38.7(774)
Other	4.4(89)
*Ever felt the need to seek help for mental distress*	
Yes	36.3(726)
No	62.2(1245)
Do not wish to answer	1.5(31)
*Ever felt the need to seek help for alcohol problems*	
Yes	4.1(83)
No	95.2(1906)
Do not wish to answer	0.6(13)
*Ever felt the need to seek help for drug use*	
Yes	2.0(41)
No	97.4(1949)
Do not wish to answer	0.6(12)
*Use of social media*	
Non-problematic	95.0(1901)
Problematic	5.0(101)
*Experience of frequent symptoms of social media addiction (endorsing options ‘often’ or ‘very often’ on BSMAS items)*	
Salience	18.1(363)
Tolerance	8.9(179)
Mood modification	6.0(120)
Failure to cut down	4.9(100)
Withdrawal	4.0(82)
Negative consequences	3.8(77)
*Time spent in instant messaging services online (hours per day)*	
Less than 1	61.2(1226)
1–2	22.2(445)
2–3	9.0(181)
3–4	4.0(80)
More than 4	3.5(70)
*Scoring of scales[range]*	Median *(interquartile range)*
PRIUSS*[0–12]*	2 *(0–4)*
GAS *[7–35]*	7 *(7–11)*
PGSI[0–27]	0 *(0–0)*
Kessler-6[0–24]	4 *(1–8)*

Given the previous literature on likely associations between social media behavior and substance use, the study included items describing problematic substance use. These variables included one dichotomous item about whether the respondent had ever felt the need to seek treatment for alcohol problems, and one variable about having felt the need to seek treatment for drug problems (defining drugs as illicit drugs and addictive prescription drugs such as prescription sedatives or strong analgesics). Also, a corresponding item assessed whether respondents had ever felt the need to seek treatment for mental distress. These items included the possibility to refuse to answer. Moreover, the quantity of time spent on online messaging services was included, with examples of such use being instant messaging services such as Facebook Messenger, Instagram Direct Messaging, as well as WhatsApp and regular phone texting. Here, options ranged from below one hour daily, to more than four hours daily (Table 1).

### Statistical methods

SPSS was used for statistical observations and analyses. A total of 2118 questionnaires were initiated, of which 116 were excluded because of incomplete status. Subsequently, descriptive characteristics were observed, and the primary outcome was characterized as problematic or non-problematic use of social media. In a first univariate comparison of respondents with problematic or non-problematic social media use, these groups were compared using the chi-square test for categorical data, and the Mann-Whitney U-test for continuous variables. Thereafter, in order to adjust variables for one another, logistic regression analyses were performed.

In a binary correlation matrix run for each pairwise combination of variables, some variables displayed relatively pronounced correlations; the association reaching the highest level of correlation (a Pearson correlation of 0.65) was between gaming (GAS) and gambling (PGSI), and three further associations were above a correlation of 0.50 (PRIUSS and GAS, 0.56, the Kessler score and having felt a need to seek treatment for mental health, 0.54, and PRIUSS and the Kessler score, 0.53). Time on instant messaging services and PRIUSS had a correlation of 0.43, and PRIUSS and PGSI 0.41, age and GAS 0.40, age and the Kessler score 0.40, and GAS and time on instant messaging 0.40, whereas all other correlations were below 0.40. Thus, in the logistic regression, data for behavioral addictions (PRIUSS, GAS and PGSI) were not assessed, due to the high statistical overlap between them, and these were therefore only reported in univariate analyses. Finally, in addition, due to the close correlation (and conceptual similarity) between the Kessler-6 score and the item describing the need to seek help for mental health, only the Kessler-6 score was included in the logistic regression. Finally, these regression analyses therefore included age group, gender, occupational status (working vs not working), level of education, number of hours spent in instant messaging services, the Kessler-6 score for mental health, and ever having felt a need to seek treatment for alcohol problems, and for drug problems, respectively). The adjusted logistic regression analyses carried out included one for the whole sample (n = 1,863, after exclusion of 139 individuals with missing data for any of the variables assessed), and one including only the youngest age groups (>40 years, n = 677, after exclusion of 59 cases with missing data).

## Results

### Descriptive observations

The number of initiated questionnaires was 2118, of which 116 were not completed and thus not included in statistical analysis. Of the remaining 2002 entries, demographics were distributed almost evenly regarding gender (1009 female, 989 male). Age distribution was skewed with the youngest groups being in minority compared to the older ones (Table 1). Educational level was split into five categories, with distribution percentages (%) presenting at 5.5 for primary school, 34.7 for high school, 16.6 for an incomplete university degree, 38.7 for a complete degree, and lastly 4.4 per cent for the category labeled ‘Other’. Having experienced a need for help seeking was reported by 36.6, 4.2 and 2.1 percent for mental distress, alcohol problems, and drug problems, respectively.

Five percent of the total sample reached scores of 19 or above of the BSMAS, indicating problematic use of social media. Of the six symptomatic components of the BSMAS scale, the most common complaint in the sample was salience (18.1%), the least common being usage leading to negative consequences (3.8%). A majority of individuals spent up to two hours (83.4%) communicating with instant messaging services, with 16.6 percent spending two hours or more. The medians for the PRIUSS, GAS, PGSI, and Kessler-6, were 2, 7, 0, and 4 respectively. A history of having felt the need to seek help for mental distress, and alcohol and drug use, were reported by 37, four and two percent, respectively.

### Associations with problematic use of social media

The univariate analysis showed no significant difference between men and women in regards to problematic use of social media, while there was a clear difference across age groups, with the highest percentages of problem users found in the younger groups (16–39 years), and a steep decrease was observed in the two older groups (40 years and above). Working or not revealed no significant difference, while educational level did, with individuals finishing high school having the highest percentage of social media addiction (7.2%), while the category of ‘other’ education showed the lowest (1.1%). Time spent chatting, as well as medians for each psychometric scale measured, were significantly associated with problematic use of social media (Table 2).

**Table 2:** Variables potentially associated with problematic social media use, %(n), unadjusted analyses. All individuals included (N = 2,002).

Variable	Problematic use (n = 101)	Non-problematic use (n = 1901)	Significance level
Age			*<0*.*001*
16–19	20.3(13)	79.7(51)	
20–24	17.8(21)	82.2(97)	
25–29	12.5(27)	87.5(168)	
30–39	7.5(27)	92.5(335)	
40–49	2.0(10)	98.0(480)	
50 or above	0.8(6)	99.2(770)	
Gender			*>0*.*10*
Female	5.7(58)	94.3(952)	
Male	4.3(43)	95.7(947)	
Occupational status			*>0*.*10*
Working	5.0(60)	95.0(1137)	
Not working	5.1(41)	94.9(764)	
Education level			*<0*.*05*
Primary School	5.4(6)	94.6(105)	
High School	7.2(50)	92.8(645)	
Incomplete Degree	4.2(14)	95.8(319)	
University Degree	3.9(30)	96.1(744)	
Other	1.1(1)	98.9(88)	
Time spent in instant messing services			*<0*.*001*
Less than 1 hour	0.7(8)	99.3(1218)	
1–2 hours	4.9(22)	95.1(423)	
2–3 hours	15.5(28)	84.5(153)	
3–4 hours	27.5(22)	72.5(58)	
More than 4 hours	30.0(21)	70.0(49)	
Scales with scoring range	Score medians (interquartile range)		
PRIUSS*[0–12]*	7 (5–9)	2 (0–4)	*<0*.*001*
GAS*[7–35]*	15 (9–25)	7 (7–10)	*<0*.*001*
PGSI[0–27]	0 (0–15)	0 (0–0)	*<0*.*001*
Kessler-6[0–24]	10 (7–14)	3 (1–7)	*<0*.*001*
Ever felt the need to seek help for…			
mental distress			*<0*.*001*
Yes	8.8(64)	91.2(662)	
No	2.7(34)	97.3(1211)	
alcohol problems			*<0*.*01*
Yes	12.0(10)	88.0(7.3)	
No	4.6(87)	95.4(1819)	
drug problems			*<0*.*001*
Yes	17.1(7)	82.9(34)	
No	4.6(90)	95.4(1859)	

Continuous variables, i.e the scales, were tested for significance through a Mann-Whitney U analysis, while categorical data were treated with chi-square testing. The 25^th^ to 75^th^ percentile signify the interquartile range.

In logistic regression, age displayed a negative associations with problematic social media use (OR 0.66 [0.55–0.78]), whereas the level of time spent in instant messaging services (OR 2.15 [1.79–2.58]), as well as the Kessler-6 score (OR 1.11 [1.05–1.17]), were positively associated with problematic social media use. Occupational status, level of education, as well as having felt a need to seek help for alcohol and drug use were not significantly correlated to the outcome (Table 3). In the second logistic regression, including only respondents below 40 years of age, the association with age was no longer statistically significant, whereas the outcome variable remained significantly associated with time using instant messaging services (OR 2.03 [1.65–2.49]), and with the Kessler-6 score (OR 1.09 [1.03–1.16], Table 4).

**Table 3:** Logistic regression analyses, associations with problematic use of social media as the dependent variable (n = 1,863). Analyses after exclusion of 139 cases with non-complete for included variables.

Variable	Odds Ratio	CI 95%
Age*	0.66	0.55–0.78
Male gender	0.75	0.45–1.27
Working	1.25	0.74–2.13
Level of education**	1.10	0.85–1.43
Time spent with instant messaging services*	2.15	1.79–2.58
Need to seek help for mental health	1.20	0.68–2.10
Need to seek help for alcohol problems	1.61	0.59–4.40
Need to seek help for drug problems	0.75	0.22–2.54
Kessler-6 score*	1.11	1.05–1.17

*p<0.05.

**Education level was analyzed covariately within the variable, omitting the category ‘other’ to rank the different education types.

**Table 4:** Logistic regression analysis, analyzing correlated of problematic social media use. Age groups 39 years and younger (N = 677). Analyses after exclusion of 59 individuals with incomplete data for included variables.

Variable	Odds Ratio	CI 95%
Age	0.84	0.62–1.14
Male gender	0.97	0.53–1.74
Working	1.08	0.59–1.99
Level of education	0.98	0.72–1.33
Time spent with instant messaging services*	2.03	1.65–2.49
Need to seek help for mental health	1.07	0.57–1.98
Need to seek help for alcohol problems	1.80	0.60–5.38
Need to seek help for drug problems	0.61	0.16–2.27
Kessler-6 score*	1.09	1.03–1.16

*p<0.05.

**Education level was analyzed covariately within the variable, omitting the category ‘other’ to rank the different education types.

Among measures of behavioral addiction, the problematic social media group had markedly higher scores for both problem internet use (PRIUSS, p<0.001), problem gaming (GAS, p<0.001), and problem gambling (PGSI, p<0.001). In addition, individuals with problematic social media use were markedly more likely to score above cut-off for problem internet use (29% vs 1% in the non-problem social media group, p<0.001), problem gaming (38% vs 3%, p<0.001), and problem gambling (56% vs 5%, p<0.001). Problem gambling, problem gaming and problem internet use were closely linked to one another; the risk of meeting established criteria of problem gambling was markedly increased with an increasing GAS score (OR 1.45 [1.38–1.52], p<0.001), or an increasing PRIUSS score (OR 1.73 [1.59–1.88], p<0.001).

## Discussion

In the present study, using a web survey addressing the general population, problematic use of social media was significantly associated with younger age, time using instant messaging, and general mental distress, and, in unadjusted analyses, also with each of the behavioral addictions including problem internet use, problematic gaming, and problematic gambling,. No independent associations were found for gender, educational level, occupational status, or having felt a need to seek help for drug or alcohol problems. The is one of few studies hitherto examining the symptoms of problematic social media use, and its correlates, in the population.

Gender did not turn out to be an independent correlate of problematic social media use. The lack of an association with female gender, when adjusting for a number of variables, is in contrast to the findings of previous studies [3, 31], although one comprehensive review [1] gathered a few studies demonstrating an association with male gender, or no association to gender at all. Speculations on the tendency of female over-representation often refer to what type of online activities are engaged in by the two genders. Women spend more Internet time on social media [30], but tend to use it for mainly for communication purposes and maintenance of already established real life relationships [23]. Conversely, results show that even though they too spend a lot of time on social media sites, men use the Internet more for gaming or gathering information compared to women, and when they engage in social media, they use it for forming new relationships and seeking communities with similar interests [23, 62].

Age was inversely correlated with problematic use of social media addiction, an observation supported by previous research [1, 3, 23]. This unsurprising find might be derived from several factors. In contrast to the childhoods of older generations, children today are increasingly exposed to technology during their formative years, at an age that seems to be steadily decreasing [27, 28]. They are taught in schools and at home how to handle technologies otherwise regarded as complex to introduce to older people, who rely more on traditional means of communication, as well as managing work and everyday life.

As children grow into teen age, they can experience increased peer pressure and a stronger need to achieve a sense of community, belonging and identity [15], which can evidently be satisfied by the various services of social media [3]. Furthermore, the fear of missing out has been proposed as another motivator for youths to constantly check their social media applications, adding to the need of constant online presence [63]. Although it remains to be seen whether higher age will be a protective factor for the addictive use of technology in the future, this current finding can be considered expected and in line with previous literature. However, despite the overall idea that problematic social media behavior may be more pronounced in the young, in the present sub-analysis of younger individuals only, the same correlates of problematic social media use remained statistically significant, except that within this narrower age group, age itself was no longer a significant correlate of the outcome variable.

Our data shows that educational level was not related to problematic use of social media, where all education categories, including the alternative ‘Other’, were separately tested against the first category, i.e. primary school. Andreasen and colleagues [23] demonstrated a relationship with lower level of education, but argued that it might be due to age rather than the education level itself. Despite the categorical features of the variable used here, and the weakness of a non-negligible number of respondents answering the alternative ‘other’, the results can be regarded as consistent with the findings of Andreasen and colleagues, although more research may be needed in order to fully shed light on the potential link between level of education and social media use. Moreover, our results showed no associations between problematic social media use and occupational status. To the researchers’ knowledge, this is an under-researched area, and findings may so far be hard to interpret.

Our study explored the relationship of problematic use of social media in relation to three commonly discussed non-substance-related addictive behaviors; problematic internet use, problem gaming, and problem gaming. Here, the analyses of the present paper are limited by the fact that these variables, between one another, tended to be too closely correlated for them to fit into the same logistic regression analysis. In particular, this holds true for the link between gambling and gaming, which displayed a relatively high degree of inter-correlation. However, it was evident that the outcome measure assessed in the present study was closely related to each of these three behavioral addictions.

For example, problematic use of social media was closely associated with the score of the Gaming Addiction Scale. In a similarly designed study, Karlsson and colleagues showed that there was a relationship between problematic gaming and addictive use of the Internet as a whole, measured by the GAS and PRIUSS scales [38], as in the present study. Despite not explicitly measuring use of social media, those results are somewhat comparable to the ones of this paper, since visiting social media sites is highly common while online [8]. Gaming and social networking may be rewarding to people in partly the same way; both channels share a potential to provide users with instant gratifications, a sense of purpose and identity, as well as satisfying social needs through online interaction [64]. Players can instantly message and talk with one another, as well as creating and being part of communities, motivating some players to use games primarily for social fulfillment. Also, probably, both gaming and social networking may even fill a purpose of personal or professional development, such as through a sports career in e-sports, or the professional and commercial value of marketing in both sectors. For example, the use of social media for professional development may apply to sectors as diverse as health professionals [65], or even information or disinformation in public health issues [66]. Likewise, gaming may range from a recreational habit to a professional career path, such as in e-sports [67]. Adding to the question of gender differences, research has shown that young men use social media sites to play games provided there, sometimes in excess, possibly making the boundaries between risky social media behavior and problematic gaming less obvious.

In addition, the present study indicated that problematic use of social media and problem gambling may correlate with one another. To the researchers’ knowledge, this is the first study assessing this relationship, thus making comparison with previous results somewhat challenging. There are however studies indicating a relationship between gambling and other technological addictions such as problematic mobile phone use, gaming and general Internet addiction [38, 68]. Some scholars have noticed similarities in design between certain aspects of casinos and social media sites. Many gambling types deliver rewards at variable ratios, a psychological mechanism notorious for being the most reinforcing type of conditioning, therefore regarded as highly addictive [69]. An example of this is slot machines, which deliver rewards at irregular intervals, making gamblers unknowing of when to expect cash payback, but expecting it nonetheless. The same can be said about some aspects of technology. The buzz of the smartphone in one’s pocket could be rewarding, a Facebook notification could be a like or a reply to a comment, but is usually something irrelevant. Together with infinite scroll, a function automatically loading more content, these functions may contribute to the addictive mechanisms of social media.

With both problematic gaming and gambling being associated with problematic use of social media, this study indicates that behavioral addictions are connected. It further supports the idea that some individuals might have a general tendency, though not always willingly or voluntarily, to do things in excess and suffer as a consequence.

Although not explicitly asking whether the individual has symptoms of addiction, the questions on having felt the need of seeking help for alcohol or drug use showed no associations with problematic use of social media. These observations may be seen as somewhat surprising, as other studies [40–42, 45] have reported associations between misusing these substances with regular and the problematic use of smartphones, the Internet and social media, findings supporting the general idea of an interrelationship between addictive conditions. The present study’s variables were brief, in the context of a web survey, and could therefore, at best, be considered a brief screening for substance use problems. Still, however, more research may be needed in order to highlight whether a problematic social media behavior may demonstrate an unexpectedly negative association with more traditional types of addictive behaviors, and importantly, this will require more in-depth screening or diagnostic instruments.

Even though addictions and mental distress are often seen together, it cannot be said with certainty where one issue starts and the other one ends. They may be different in their expressions, but could they share some core origin, a primal human drive for a sense of purpose and belonging? Addressing problematic social media use as a facet of mental health issues could help health care professionals treat their patients synergistically from multiple angles. For example, while an association was demonstrated between problematic social media use and poor mental health, this association does not imply causality. As a large review on Facebook-related depression studies demonstrated, the strongest predictor of depression was not the time spent on Facebook or how frequently it was checked for updates, but rather how much the user compared their life situation with the appearances and activities of others [70]. This could possibly create and strengthen any perceived social and cultural pressure to be happy and successful, bringing more shame over one’s cognitions, emotions and behaviors.

Moreover, it can be used to strengthen the screening for problematic social media habits in clinical settings where patients with mental health problems are assessed, and likewise, in case of an assessment for an exaggerated social media use, an individual’s mental health should be assessed. Thus, even while demonstrating only cross-sectional associations without further evidence of causality, there may be implications of relevance for routine clinical settings.

Five percent of our total sample had a score of 19 or higher on the Bergen Social Media Addiction Scale, indicating problematic social media use. One interesting finding was the distribution of component symptomatology of social media use. By observing what proportions of our sample answered ‘Often’ or ‘Very often’ on any item, we saw that 18.1 percent were highly preoccupied with social media. This might be affected by several factors, one being that scales measuring addictions generally go from mild to severe experiences, where sole preoccupation is rarely seen without any other symptoms or experiences in addictions. Another factor could be that social needs are increasingly fulfilled online, and just as real-life social situations, people go about their days thinking a lot about what they said to somebody or what they are planning to do with friends, without substantial complaints. Adding to this, the question was phrased as how much time one spent thinking about social media, not how much they worried about it.

The uneven proportion of social media related experiences might indicate that some components contribute more to a state of suffering and addiction than others. This is reflected in the less reported withdrawal symptoms and social media use leading to adverse consequences in real life, whereas the item describing salience was markedly more commonly reported. Such experiences are especially relevant in the context of mental health, where some symptoms emerge as more probable contributors, maintainers and consequences of mental unease. Suffering frequent withdrawal symptoms such as anxiety and irritability might increase existent mental distress. If the individual then finds these moods being alleviated through social media, the component of mood modification emerges as an important element. For individuals without manifest mental disorders, times of stress and tension can lead to escapism for the same reasons. If psychosocial needs are better met online, addiction can manifest as an overreliance on virtual interactions. The difficulty to control use, or stopping it completely, may contribute to general stress and feeling a lack of free will, as well as shame and guilt, two common features of depression. These mind states could in turn be distracted from, but also exaggerated by, using social media. Anxious people may resort to Facebook, Instagram, Snapchat or YouTube if it can provide them with more control over social situations. For example, choosing with greater freedom who to interact with, or dare to express opinions more readily, especially if their anxiety revolves around social situations. Be it depression or anxiety, symptoms might be further exacerbated should social media absorb enough time and energy to damage relationships, work or studies, a potentially serious and debilitating consequence of any addiction. Naturally, the magnitude of these consequences is likely to be smaller than those seen in severe addictions to gambling or substances.

Although there was a significant difference between the groups studied with respect to the amount of time spent communicating in instant messaging services, our results also show that the majority of those who spent more than four hours chatting did not qualify for problematic use. Naturally, this is not equal to them never experiencing distress. However, it indicates that users might well use online chats as their main channel of communication, and in that context, the terminology of ‘addiction’ may present a risk to pathologize and stigmatize common behaviors, especially when they consume a lot of time. Among its many young users, a number of benefits are reported, such as a sense of belonging, establishment of identity, learning skills, as well as improve relationships with virtual and real-world friends. Moreover, attitudes differ between technology when used excessively by children; they themselves regard it as less of a problem or an addiction for that matter, compared to their parents who more often see screen time as negative [3]. In a debate paper, Kardefeldt and co-workers argue that many behaviors can be performed in excess without being perceived as addictive for the individual. Working eight hours per day is commonplace and can come out of necessity and duty, enjoyment and a sense of purpose, rather than escapism, compulsiveness, or cravings. Professionals of arts or sports, including e-sports, may well compromise other life areas or experience distress when unable to do what they like [10], although the concept of an addictive condition may be less applicable to these situations.

Also utilizing the BSMAS, in their large study Andreassen and co-workers found that excessive use of social media was linked to symptoms of ADHD and OCD, as well as narcissistic and extraversion personality traits [23, 29]. Even though these respondents scored higher on the social media addiction scale, it does not necessarily translate into addiction. The paths to excessive use and addiction-like behavior are thought to be different for people with these traits, with decreased executive functioning or compulsiveness leading to loss of the control that technology has on these individuals, while they might not experience cravings or withdrawal. Such potential associations also should be borne in mind when assessing whether a description of an addiction is appropriate or not.

### Strengths and limitations

Most empirical studies into addictive technological behaviors rely on small and/or non-representative samples [25, 71–73], often surveying students form primary school to university. The sample used in the present study was moderately sized, comprising 2002 respondents including individuals of 16 years and above. However, our sample has a skewed age distribution and is therefore not completely nationally representative, despite an overall ambition to reach representativity. Consequently, any conclusions for prevalence rates of problematic social media cannot be made from our results.

An obvious limitation of the present finding is the fact that all data were self-reported, and although using established scales for the measure of several constructs included here, the self-report of several measures means that the individual’s own perception of one’s behavior may affect the reporting. This could, for example, apply to the reporting of the number of days spent in social messaging services per day; a person with an impression of being more preoccupied than desired with these services, could possibly lead to lower her/his reporting.

Using a market survey company made it possible to disidentify respondents’ IP-addresses, while simultaneously enabling detection of duplicates. Had the same respondent filled the questionnaire more than once from different IPs, the design could not account for that. The length of the questionnaire may, however, reasonably make such an attempt unappealing.

The choice to omit some variables is motivated by initially weak hypotheses, and retrospectively discovered correlational overlap. The reason for including the demographic variables of gross monthly income and living situation is mostly that previous studies carried out by the same research department as this study have included them as well. One could argue that they needed not to be part of the questionnaire only for this reason. Seeking help for mental distress and PRIUSS scoring were found to skew the correlational data of the logistic regression analyses, which might question the choice to include them from the beginning.

One flaw of the addiction scales is that they assess symptomatology for different time frames, rendering them less intercomparable to each other. They range from the past 30 days to the past 12 months, and while PRIUSS is measuring momentaneous experience of Internet use, it is a factor not taken into consideration since it was omitted from regression analysis. The scales are validated and cross-validated across languages, justifying them as tools and comparing them with other studies using them. Naturally, comparing with results stemming from other tools is more speculative. An agreed upon research method would diminish this issue.

It is important to note that all scales are screening tools and not diagnostic instruments, meaning that the demonstrated relationships are based on scores and cutoffs of these scales. Therefore, it is not possible to draw conclusions on if, and to which degree the actual *diagnoses* co-occur. While this is a limitation of the study with respect to the lack of formal diagnostic information, the use of brief screening tools was based on the need to maintain a relatively brief total survey content, in the format of an online survey.

### Study implications

Defined treatment strategies specifically targeting social media addiction are lacking, although extensive work by Young [49] laid a fundament for cognitive behavioral therapy for general Internet addiction. Since neither the Internet nor social media can reasonably be taken away from people, abstinence protocols such as those used for substances cannot reasonably be applied. Rather, approaches focus on restriction. Managing Internet use in general has traditionally equaled to self-blocking of various websites more prone to addiction, such as pornography or shopping sites. In a social media context, this kind of selective restriction may be hindered by the fact that potentially beneficial and dangerous services exist on the same web page. Limiting access to specific functions on Facebook, Instagram or any other platform might hopefully be enabled by the companies themselves or third parties in the near future. In fact, some appliances have already implemented regulators of use such as app-stop timers, screen time tracking, and proposals to remove the infinite scroll function as well as limiting like-buttons have been made.

Being that problematic use of social is associated with mental distress as well as with problematic gaming and problem gambling, this could motivate health care professionals meeting patients suffering from these associated disorders to also ask them about their social media habits and experiences. One implication is that more research is needed in order to establish possible causality, which cannot be concluded from the present study, but still, there may be implications for routine screening and assessment in clinical settings where any of these conditions are treated, and where other potentially addictive behaviors, or poor mental health, also may be present.

## Conclusions

The present study demonstrated a complex interrelationship between problematic use of social media and mental distress, with time spent in instant messaging services, as well as with other behavioral addictions, but not with a history of treatment needs for substance use. Young age was also associated with problematic social media use, while no associations were observed for educational level or occupational status. Conclusions cannot be drawn for the general population as our sample was somewhat skewed in regard to age. Since the measuring tools used are for screening purposes, we cannot conclude whether any corresponding disorders are related either, but rather that higher scores on the scales are seen with one another.

To further expand the emerging field of technology-related addictions, agreed upon definitions and measurement tools, as well as more studies are needed. With longitudinal data, it will be very interesting to see how the use, abuse, or restricted use of social media will contribute to functioning and perception of the individual. Because of their early exposure and norms of technology, observing young generations as they age would be especially fascinating. Given the results of the present study, mental health and social media use will be important to detect and follow in preventive work and in clinical settings.

## Supporting information

S1 File (PDF)

S2 File (PDF)
