# Supplementary material for: Hooked on virtual social life. Problematic social media use and associations with mental distress and addictive disorders
Source: PLoS One. 2021 Apr 8;16(4):e0248406. doi: 10.1371/journal.pone.0248406 (PMC8032197; doi:10.1371/journal.pone.0248406)
Supplement: S1 File — (PDF) [file pone.0248406.s001.pdf]

<u>Translation of attached decision from the Swedish Ethical Review Authority (file number 2019-04176)</u>

Applicant organization

Lund University

Researcher conducting the project

Anders C Håkansson

Project title

Screen- and gambling-related addiction in the Swedish population – significance and association with behavioral addictions

---

The Ethical Review Authority decides as below: The Ethical Review Authority also provides an advising statement according to 4a§ law (2003:615) of ethical review of research concerning humans.

DECISION

The Ethical Review Authority does not try the application for approval.

Reasons for decision

The project does not handle sensitive personal data. The project also is not of such a nature that it is covered by the law (2003:460) of ethical approval regarding research in humans. Therefore, the Ethical Review Authority cannot try the application for approval.

The Ethical Review Authority does not have any ethical concerns regarding the research project.

I hereby confirm that the translation above is a direct translation of the attached decision by the Swedish Ethical Review Authority.

Lund, Sweden, March 18th 2020

Åsa Westrin, consultant physician, professor, head of Division of Psychiatry, Dept of Clinical Sciences Lund, Faculty of Medicine, Lund University
asa.westrin@med.lu.se
