# Supplementary material for: Hooked on virtual social life. Problematic social media use and associations with mental distress and addictive disorders
Source: PLoS One. 2021 Apr 8;16(4):e0248406. doi: 10.1371/journal.pone.0248406 (PMC8032197; doi:10.1371/journal.pone.0248406)
Supplement: S2 File — (PDF) [file pone.0248406.s002.pdf]

# BESLUT

Dnr: 2019-04176

2019-08-14

**Sökande forskningshuvudman**

Lunds universitet

**Forskare som genomför projektet**

Anders C Håkansson

**Projekttitel**

Skärm- och spelberoende i den svenska befolkningen - betydelse av och samband med beteendeberoenden

---

Etikprövningsmyndigheten beslutar enligt nedan. Etikprövningsmyndigheten lämnar samtidigt ett rådgivande yttrande enligt 4 a § förordningen (2003:615) om etikprövning av forskning som avser människor.

**BESLUT**

Etikprövningsmyndigheten tar inte upp ansökan till prövning för godkännande.

**Skäl för beslut**

Projektet innebär inte någon behandling av känsliga personuppgifter. Projektet är inte heller i övrigt av sådan art att det omfattas av lagen (2003:460) om etikprövning av forskning som avser människor. Etikprövningsmyndigheten kan därför inte ta upp ansökan till prövning för godkännande.

Etikprövningsmyndigheten har inte några etiska invändningar mot forskningsprojektet.

---

Beslutet att inte ta upp ansökan till prövning kan överklagas hos Överklagandenämnden för etikprövning. Hur man överklagar framgår av bifogad anvisning. Det rådgivande yttrandet får inte överklagas.

---

På Etikprövningsmyndighetens vägnar

Christian von Szalay

Ordförande

---

Vid beslutsfattandet har följande personer medverkat;

**Ordförande**
Christian von Szalay

**Ledamöter med vetenskaplig kompetens**
Christina Hultman, föredragande
Maria Feychting (Miljömedicin, epidemiologi), Ledamot
Olof Sköldenberg (Ortopedi), Ledamot
Miriam Mints (Gynekologi), Ledamot
Göran Erik Elinder (Pediatrik), Ledamot
Claudia Lampic (Vårdvetenskap), Ledamot
Erik Näslund (Kirurgi), Vetenskaplig sekreterare

**Ledamöter som företräder allmänna intressen**
Bo Bängtsson, Ledamot
Gunilla Thorsson, Ledamot

---

**Beslutet sänds till:**
Ansvarig forskare: Anders C Håkansson
Forskningshuvudmannens företrädare: Mikael Bodelsson

## Hur man överklagar Etikprövningsmyndighetens beslut

**Vem får överklaga?**
Det är forskningshuvudmannen som får överklaga Etikprövningsmyndighets beslut om det har gått sökanden emot. Överklagandet ska vara skriftligt. Skrivelsen ska vara undertecknad av behörig företrädare för forskningshuvudmannen.

Om forskaren överklagar ska en fullmakt från forskningshuvudmannen bifogas.

**När ska beslutet senast överklagas?**
Överklagandet ska ha kommit in till Etikprövningsmyndigheten inom tre veckor från den dag då forskningshuvudmannen fick del av beslutet.

**Vad ska överklagandet innehålla?**
Överklagandet ska innehålla uppgifter om

1. klagandens namn, person- eller organisationsnummer, adress, telefonnummer och e-postadress
2. det beslut som överklagas (dag för beslut, projekttitel och diarienummer)
3. hur ni anser att nämndens beslut ska ändras och skälen till att beslutet bör ändras.

**Var ska överklagandet skickas?**
Överklagandet ska ställas till Överklagandenämnden för etikprövning. Men det ska skickas eller lämnas till Etikprövningsmyndigheten.

Om överklagandet har kommit in i rätt tid överlämnar Etikprövningsmyndigheten överklagandet och handlingarna till Överklagandenämnden för etikprövning.
